# Multidimensional Effects of Suryanamaskar on Physical, Physiological, and Psychological Outcomes: A Systematic Review

**DOI:** 10.3390/healthcare14131924

**Published:** 2026-07-01

**Authors:** Suchishrava Choudhary, Prashant Kumar Choudhary, Sohom Saha, Nicolae Ochiană, Bogdan Alexandru Antohe, Cristina Ioana Alexe

**Affiliations:** 1 Department of Physical Education Pedagogy, Lakshmibai National Institute of Physical Education, Gwalior 474002, Madhya Pradesh, India; suchishrava05@gmail.com (S.C.); prashantlnipe2014@gmail.com (P.K.C.); 2 Department of Physical Education, Mahatma Gandhi Government College, Pondicherry University, Mayabunder 744204, North and Middle Andaman, India; 3 Department of Sport Psychology, Lakshmibai National Institute of Physical Education, Gwalior 474002, Madhya Pradesh, India; sohomsaha77@gmail.com; 4 Faculty of Movement, Sports, and Health Sciences, “Vasile Alecsandri” University of Bacău, 600115 Bacău, Romania; sochiana@ub.ro (N.O.); antohe.bogdan@ub.ro (B.A.A.)

**Keywords:** suryanamaskar, yoga intervention, physical fitness, physiological adaptations, psychological well-being

## Abstract

**Background:** Suryanamaskar (Sun Salutation) is a dynamic sequence of yoga that incorporates movement, breath and mindfulness, and is known for its many potential multidimensional health benefits. Despite the increasing volume of research, a comprehensive and domain-specific synthesis examining the multidimensional effects of Suryanamaskar and yoga-based interventions incorporating Suryanamaskar remains limited. Hence, the present study was designed to systematically review and synthesize the existing evidence related to the effects of Suryanamaskar and yoga-based interventions using Suryanamaskar sequence on various populations and outcome domains. **Methods:** This systematic review was conducted in accordance with the PRISMA 2020 statement and Cochrane Handbook recommendations. Literature searches were performed in PubMed, Scopus, Web of Science, and Google Scholar from database inception to 31 December 2025. Studies involving human participants and Suryanamaskar-based interventions reporting measurable physical, physiological, or psychological outcomes were included. Fourteen studies met the eligibility criteria. Study characteristics, intervention protocols, and outcome measures were extracted. Risk of bias was assessed using RoB 2 for randomized studies and ROBINS-I for non-randomized studies. Due to substantial heterogeneity, findings were synthesized narratively. **Results:** A total of 14 studies met the inclusion criteria. Overall, Suryanamaskar and yoga-based interventions incorporating Suryanamaskar were associated with improvements in physical fitness, physiological health, psychological well-being, and body composition across diverse populations. Most included studies reported favourable changes in physical fitness, physiological, psychological, and body-composition outcomes; however, the magnitude and consistency of findings varied substantially across study designs, participant populations, intervention protocols, and outcome measures. **Conclusions:** Promising but heterogeneous evidence suggests that Suryanamaskar and yoga-based interventions incorporating Suryanamaskar may contribute to improvements in physical fitness, physiological function, psychological well-being, and body composition across diverse populations. However, these findings should be interpreted with caution because of variability in study designs, intervention protocols, participant characteristics, and risk of bias. Although Suryanamaskar appears to be a practical, low-cost, and holistic intervention with potential applications in educational, sports, and health-promotion settings, further high-quality randomized controlled trials with standardized protocols and larger sample sizes are required to strengthen the evidence base.

## 1. Introduction

Suryanamaskar (Sun Salutation) is the structured sequence of yogic postures that are performed in a cycle with the synchronized movement patterns and breathing [1,2]. Its benefits on the physical, physiological and psychological aspects of health have made it a widely adopted exercise, which has grown out of the traditional yoga systems [2,3]. This series is usually composed of 10–12 postures where multiple muscle groups and body systems are engaged in multiple transitions to forward and backward bending, stretching and weightbearing, together [4,5].

Suryanamaskar is a distinctive and effective full body exercise intervention that allows combined strength, flexibility, endurance, and coordination exercises to be performed in a single continuous session [1,6]. Physiologically, Suryanamaskar is linked to substantial enhancements in cardiovascular, respiratory and autonomic functions. Controlled breathing and the dynamic nature of the sequence maximize the amount of oxygen used and thus energy utilization is optimized [7,8]. Yoga has been shown to reduce autonomic imbalance, increase parasympathetic activity and improve baroreflex sensitivity, leading to better stress regulation and cardiovascular health [9,10]. Moreover, regular exercise has been shown to impact metabolic parameters, such as weight loss and decreases in body mass index, body fat percentage, and waist circumference, which suggests its importance in obesity management and related conditions [11,12]. These physiological changes indicate Suryanamaskar as a modality that brings integrative functioning of the internal systems.

From a physical fitness perspective, Suryanamaskar represents a comprehensive exercise modality capable of enhancing muscular strength, endurance, flexibility, balance, and neuromuscular coordination through the integration of dynamic postural transitions and weight-bearing movements. The repetitive and weight-bearing postures facilitate strengthening of the upper and lower body strength and the stretching component increases the mobility and flexibility of the joints and musculoskeletal system [13,14,15]. Moreover, the synchrony of the movement between postures calls for neuromuscular control and stability, which enhances balance and proprioceptive skills [16]. The combination of these effects makes Suryanamaskar a good physical conditioning system which can be used in various groups such as athletes and non-athletes.

A growing body of evidence has demonstrated the potential psychological benefits of Suryanamaskar and related yoga-based interventions, particularly in relation to stress regulation, emotional well-being, and mental health outcomes. Breathing exercises, physical activity, and mindfulness are all important components of reducing stress and improving mental health. The data indicate that yoga practices have a modulatory effect on neurophysiological mechanisms related to the hypothalamic–pituitary–adrenal axis and the autonomic nervous system, resulting in decreased stress responses and enhanced emotional regulation [17,18]. Empirical studies have found that practitioners of Suryanamaskar [19,20,21] showed significant reduction in anxiety, depression and perceived stress and improvement in emotional intelligence, resilience and overall quality of life, among others. The findings support the notion of Suryanamaskar as a comprehensive mind–body technique that can impact psychological and physiological areas.

Despite the growing body of evidence, a comprehensive synthesis specifically examining the multidimensional effects of Suryanamaskar and yoga-based interventions incorporating Suryanamaskar remains limited. While several studies have investigated individual outcome domains such as flexibility, cardiovascular function, body composition, or psychological well-being, these findings have largely been reported in isolation. Furthermore, variations in intervention design, participant characteristics, training duration, outcome assessment methods, and inconsistencies in effect-size reporting have made it difficult to establish a unified understanding of the effectiveness of Suryanamaskar-related practices and have reduced the clarity of evidence synthesis [22,23]. Existing reviews have generally focused on yoga as a broad intervention or have examined selected health and wellness outcomes associated with Surya Namaskar, without comprehensively evaluating its multidimensional effects across physical fitness, physiological, psychological, and body-composition domains. Accordingly, the present review should be considered an extension and update of the existing evidence base rather than the first synthesis on the topic. The present review expands previous reviews by simultaneously examining multidimensional outcomes across diverse populations while distinguishing between standalone Suryanamaskar interventions and broader yoga-based programmes that explicitly incorporate Suryanamaskar as a defined intervention component. This approach provides a more comprehensive understanding of the potential contributions of Suryanamaskar to health, fitness, and performance outcomes.

## 2. Materials and Methods

### 2.1. Study Selection Procedures

**Figure 1:**
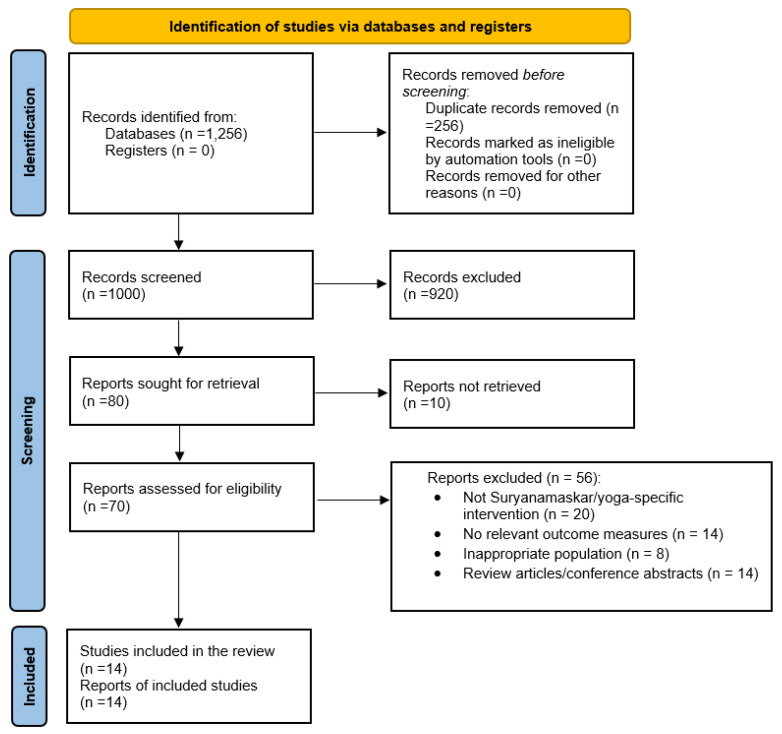
PRISMA flow diagram illustrating the study selection process.

All the steps taken to select the studies were done in line with the internationally recognized guidelines for systematic reviews, specifically Preferred Reporting Items for Systematic Reviews and Meta-Analyses (PRISMA) and the recommendations of the Cochrane Collaboration. This systematic process also ensured methodological rigour, transparency and reproducibility in the review process. All records that were found through a thorough search of the databases were first imported into a reference management program where duplication was systematically identified and eliminated. This reduced duplication and ensured that each study was only looked at once during the screening process. A two-stage screening process was conducted after deduplication. In the first stage, two reviewers independently screened the titles and abstracts of all retrieved records using the Population, Intervention, Comparator, Outcomes, and Study Design (PICOS) framework (Table 1). Studies that clearly failed to meet the eligibility criteria, including those without Suryanamaskar-based interventions, those not reporting quantitative outcome measures, and those not involving human participants, were excluded at this stage. In the second stage, the full texts of potentially eligible articles were independently assessed by the same reviewers to determine final inclusion. Any disagreements between reviewers were resolved through discussion and consensus. When consensus could not be reached, a third reviewer acted as an adjudicator and made the final decision. All disagreements identified during title/abstract and full-text screening were resolved through discussion and consensus before data extraction. This procedure was implemented to minimize selection bias and strengthen the methodological rigor, transparency, and reliability of the review process. The studies were rigorously examined and it was ensured that each study specifically examined the effects of Suryanamaskar or a yoga intervention that includes Suryanamaskar on at least one domain of interest, such as physical fitness, physiological parameters, psychological outcomes or body composition. Specific focus was placed on intervention fidelity, outcome measurement validity and study design appropriateness. Screening and eligibility assessment were done separately to increase the reliability of the selection process; any differences and disagreements were discussed and agreed upon. When necessary, guidance from the existing systematic review literature was sought to facilitate consistency for decision-making. This cooperation reduced selection bias and increased the credibility of the review. The review protocol was registered in PROSPERO (CRD420261387567) on 08 May 2026. Registration occurred after completion of the literature search and study selection procedures but before the final evidence synthesis and manuscript preparation. Accordingly, the registration is reported to enhance transparency and protocol accessibility rather than as a prospectively registered review. In addition, all of the study selection process was systematically documented, and the number of records identified, screened, excluded (with reasons), and included in the final synthesis was reported. A supplementary table detailing categories of full-text articles excluded during eligibility assessment and the corresponding reasons for exclusion is provided in Supplementary Table S1. This documentation helps in ensuring transparency and enables the process to be replicated by future researchers, following best practices in evidence synthesis [24]. A detailed PRISMA 2020 flow diagram illustrating the identification, screening, eligibility assessment, and inclusion of studies is presented in Figure 1.

### 2.2. Literature Search: Administration and Update

A detailed and systematic literature search was conducted to identify all relevant studies examining the effects of Suryanamaskar and yoga-based interventions incorporating Suryanamaskar on physical, physiological, and psychological outcomes. The search strategy was developed following the Cochrane Handbook and PRISMA guidelines to ensure methodological rigour, transparency, and reproducibility. Four major electronic databases, namely PubMed, Scopus, Web of Science, and Google Scholar, were searched. The literature search covered all eligible studies available from database inception until 31 December 2025. These databases were selected to ensure comprehensive coverage of peer-reviewed literature across biomedical, sports science, and interdisciplinary research fields. A search strategy incorporating controlled vocabulary (e.g., MeSH terms in PubMed) and free-text keywords was employed to maximise sensitivity and specificity. Boolean operators (“AND” and “OR”) were used to organize and refine the search strategy.

The search strategy was designed to maximise sensitivity while maintaining retrieval relevance. Search terms primarily focused on the intervention concept, including “Suryanamaskar”, “Surya Namaskar”, “Sun Salutation”, and related yoga terminology, combined with human-participant filters where applicable. Outcome-related terms were incorporated to improve retrieval efficiency and identify studies across a broad range of health and performance domains; however, eligibility was not restricted to any specific outcome category during study selection. Consequently, studies reporting physical fitness, physiological, psychological, cognitive, body-composition, metabolic, sleep-related, anthropometric, or other health-related outcomes remained eligible provided that they investigated a Suryanamaskar-based intervention and satisfied the predefined inclusion criteria. The following database-specific search strategies were subsequently applied: In PubMed, the search string used was: (“Suryanamaskar” OR “Surya Namaskar” OR “Sun Salutation”) AND (“Yoga”[Mesh] OR yoga OR “yogic exercise”) AND (“Physical Fitness”[Mesh] OR “Motor Activity”[Mesh] OR “Exercise”[Mesh] OR “physical fitness” OR strength OR endurance OR flexibility OR balance OR “physiological outcomes” OR “cardiorespiratory fitness” OR “VO2 max” OR “psychological outcomes” OR stress OR anxiety OR depression OR well-being) AND (Humans[Mesh]). The search strategy used in Scopus was: TITLE-ABS-KEY (“suryanamaskar” OR “surya namaskar” OR “sun salutation”) AND TITLE-ABS-KEY (yoga OR “yogic exercise”) AND TITLE-ABS-KEY (“physical fitness” OR strength OR endurance OR flexibility OR balance OR “physiological outcomes” OR “cardiorespiratory fitness” OR “VO2 max” OR “psychological outcomes” OR stress OR anxiety OR depression OR “mental health” OR well-being). The following search string was used in Web of Science: TS = (“suryanamaskar” OR “surya namaskar” OR “sun salutation”) AND TS = (yoga OR “yogic exercise”) AND TS = (“physical fitness” OR strength OR endurance OR flexibility OR balance OR “physiological outcomes” OR “VO2 max” OR “psychological outcomes” OR stress OR anxiety OR depression OR well-being). The search string used for Google Scholar was: (“Suryanamaskar” OR “Surya Namaskar” OR “Sun Salutation”) AND yoga AND (“physical fitness” OR “physiological outcomes” OR “psychological outcomes” OR stress OR anxiety OR depression). In line with systematic review methodology, only the first 200 results sorted by relevance were screened because of the wide range of Google Scholar. Because Google Scholar does not provide a direct peer-review filter, all records identified through Google Scholar were manually screened. Peer-reviewed status was verified through journal information, publisher websites, indexing databases, and article metadata during the eligibility assessment process. Only studies published in peer-reviewed English-language journals were ultimately included in the review. In addition, a manual search of the reference lists of included articles and relevant reviews was done to ensure that further articles that might not have been found in the database search were not overlooked. The search process was an iterative one, and the search was updated on a regular basis before final data synthesis was completed, to include the most updated studies, reflecting the most up-to-date evidence base. The methods used for searching (e.g., keywords, using Boolean operators, and sometimes adapting search (AS) strategies to the database) were thoroughly documented and reported, following best practice guidelines for conducting systematic reviews [24,25].

### 2.3. Data Extraction

Data extraction was carried out systematically in accordance with the recommendations of the Cochrane Collaboration and the PRISMA 2020 statement using a standardized and pilot-tested data extraction form. Two reviewers independently extracted data from all included studies, and any discrepancies were resolved through discussion and consensus. When agreement could not be reached, a third reviewer was consulted to adjudicate the final decision. Extracted information included study authors, country, study design, participant characteristics (sample size, age, and sex), intervention details (type, duration, and frequency), comparator characteristics, outcome variables, and key findings. In addition, statistical information, including mean values, standard deviations, *p*-values, and reported effect sizes, was collected whenever available. For studies that did not report effect sizes, alternative indicators such as percentage change, t-values, F-values, or descriptive interpretations were recorded to facilitate comparison of intervention effects across studies. The extracted data were subsequently cross-checked for completeness, accuracy, and consistency prior to synthesis. Several included studies were authored by members of the review team. To minimise potential bias, eligibility assessment, data extraction, and risk-of-bias evaluation for these studies were independently verified by co-authors who were not involved in the original primary research.

### 2.4. Summary Measures

The key summary variables were the change in outcome variables from pre- to post-intervention across the studies included. Changes were generally reported in terms of mean differences and standard deviations, if available. Reported *p*-values were used for statistical significance, and *p* < 0.05 was considered statistically significant. Standardised effect sizes (Cohen’s d and partial eta squared [η^2^]) were used where reported, to allow for assessment of the magnitude of intervention effects. For studies in which effect size was not reported, alternative measures such as percentage change, t or F values, or descriptive interpretations of the statistical results were used to approximate the direction and magnitude of the effects. When controlled comparisons were available, between-group effects were prioritised because they provide stronger evidence of intervention effectiveness. Within-group changes were reported descriptively to illustrate temporal changes but were interpreted cautiously, particularly in uncontrolled and self-controlled study designs where improvements may reflect factors other than the intervention itself.

### 2.5. Synthesis of Results

Considering the substantial methodological and clinical heterogeneity among the included studies, a structured narrative synthesis approach was adopted. Studies were first categorized according to four predefined outcome domains: (1) physical fitness, (2) physiological parameters, (3) psychological outcomes, and (4) body composition. Within each domain, findings were synthesized by comparing the direction of effect (improvement, deterioration, or no change), statistical significance, and reported effect magnitude where available. Greater emphasis was placed on identifying consistent patterns across studies rather than on individual study findings. The consistency of evidence was evaluated according to the proportion of studies reporting favourable outcomes within each domain, while consideration was also given to differences in participant characteristics, intervention duration, intervention composition, and study design. Because of heterogeneity in outcome measures and intervention protocols, quantitative pooling was considered inappropriate, and findings were interpreted descriptively.

### 2.6. Risk-of-Bias Assessment

The methodological quality of the included studies was assessed using risk-of-bias tools appropriate to the respective study designs. Randomized controlled trials were evaluated using the Cochrane Risk of Bias 2 (RoB 2) tool, whereas non-randomized, quasi-experimental, and self-controlled intervention studies were assessed using the Risk of Bias in Non-randomized Studies of Interventions (ROBINS-I) framework [26]. The RoB 2 assessment considered bias arising from the randomization process, deviations from intended interventions, missing outcome data, measurement of the outcome, and selection of the reported result. For non-randomized studies, the ROBINS-I framework was used to evaluate potential bias related to confounding, selection of participants into the study, classification of interventions, deviations from intended interventions, missing data, measurement of outcomes, and selection of the reported result. Because RoB 2 and ROBINS-I assess different sources of bias and employ different judgment criteria, their results were presented separately according to study design. Risk-of-bias assessments were conducted independently by two reviewers, and any disagreements were resolved through discussion and consensus. Overall methodological quality was considered during data synthesis and interpretation of findings. The assessment procedures followed recommendations outlined in the Cochrane Handbook for Systematic Reviews of Interventions and the guidance for the RoB 2 and ROBINS-I assessment tools [24,26].

## 3. Results

Overall, 14 studies met the inclusion criteria and were included in the narrative synthesis. Most studies were conducted in India and employed randomized, experimental, or quasi-experimental designs. Intervention durations ranged from 4 to 24 weeks, with frequencies between 2 and 6 sessions per week. Of the 14 included studies, eight evaluated standalone Suryanamaskar interventions, whereas six investigated yoga-based programs that explicitly incorporated Suryanamaskar as a defined component of the intervention. No included study was classified as a yoga-based intervention without a clearly identifiable Suryanamaskar component. Outcomes were broadly categorized into physical fitness, physiological parameters, psychological outcomes, and body composition (Table 2).

Table 3 presents the RoB 2 assessment of the nine randomized studies included in this review. Overall, the methodological quality of the randomized evidence was characterized predominantly by ‘some concerns’ across most bias domains. The most common issues were related to insufficient reporting of randomization procedures, allocation concealment, deviations from intended interventions, and selective outcome reporting. Most studies demonstrated adequate management of missing outcome data and generally employed validated and standardized outcome measures. However, limited methodological reporting prevented several studies from being judged as having low risk of bias. Collectively, the findings indicate that although the randomized evidence base is generally acceptable, methodological limitations and incomplete reporting procedures warrant cautious interpretation of the observed intervention effects.

Table 4 presents the ROBINS-I assessment of the five non-randomized studies included in the review. Overall, these studies demonstrated a greater susceptibility to bias than the randomized studies, with all investigations receiving an overall judgement of serious risk of bias. The most prominent sources of bias were related to confounding, participant selection procedures, and deviations from intended interventions, reflecting the inherent limitations of quasi-experimental and self-controlled study designs. In contrast, risks associated with missing data and selective outcome reporting were generally judged to be moderate, while classification of interventions and outcome measurement domains demonstrated low-to-moderate or moderate concerns across studies. Although these investigations frequently reported favourable outcomes and employed validated outcome measures, the absence of rigorous randomization procedures and the potential influence of uncontrolled confounding factors limit causal inferences regarding intervention effectiveness. Consequently, findings derived from the non-randomized evidence base should be interpreted with greater caution than those obtained from the randomized studies.

Table 5 provides a summary of the effects of Suryanamaskar and yoga-based interventions incorporating Suryanamaskar across the included studies. Overall, favourable trends were observed across physical fitness, physiological, psychological, and body composition domains. Improvements were most consistently reported for flexibility, muscular strength, endurance, balance, cardiovascular fitness, stress reduction, and quality of life. However, the magnitude of effects varied across studies because of differences in participant characteristics, intervention protocols, and outcome assessment methods.

A narrative certainty-of-evidence assessment based on GRADE principles indicated that the overall certainty of evidence was low for physical fitness and physiological outcomes and very low for psychological and body-composition outcomes, primarily because of methodological limitations, substantial heterogeneity, and the inclusion of several non-randomized studies. Detailed certainty ratings and reasons for downgrading are presented in Supplementary Table S2.

## 4. Discussion

The current systematic review included 14 studies to assess the effectiveness of Suryanamaskar and yoga-based interventions on physical, physiological and psychological outcomes in various population groups. The results frequently showed that these interventions have numerous multidimensional benefits, reinforcing their effectiveness as a whole-body exercise modality. In the studies included, we saw improvements in a variety of domains across all ages, genders and population types, indicating wide applicability. The overall findings suggest that Suryanamaskar and yoga-based interventions incorporating Suryanamaskar may contribute to improvements in human performance and health outcomes across multiple domains, although the strength of evidence varies according to study design and methodological quality. While the overall findings suggest beneficial effects of Suryanamaskar and yoga-based interventions incorporating Suryanamaskar across multiple outcome domains, the strength of evidence varied according to study design. Evidence was generally stronger in randomized and controlled studies, where between-group comparisons provided greater confidence in the observed effects. In contrast, findings from uncontrolled pre–post and quasi-experimental studies should be interpreted with caution because they are more susceptible to confounding factors, selection bias, and regression-to-the-mean effects. Furthermore, substantial heterogeneity in participant characteristics, intervention protocols, comparator conditions, and outcome measures limited direct comparisons across studies and reduced certainty regarding the magnitude of intervention effects.

### 4.1. Effects on Physical Fitness

One of the most significant findings of this review is the steady progression of physical fitness parameters including muscular strength, muscular endurance, flexibility and balance. Research in this review showed that there were strong increases in both upper and lower body strength, core stability and functional movement. Bhutkar et al. (2011) found significant improvements in strength and endurance after a structured Suryanamaskar program while Ethiraj et al. (2024) noted significant increases in spinal flexibility and Dubey and Choudhary (2024) noted significant increases in spinal flexibility [13,14,29]. Likewise, Mangaonkar and Puntambekar (2018) also reported that Suryanamaskar is more effective in enhancing hamstring flexibility than dynamic stretching [15]. The improvements have been attributed to the dynamic, repetitive, and weight-bearing nature of Suryanamaskar, which simultaneously engages multiple muscle groups and enhances neuromuscular coordination and functional movement capacity, findings that are broadly consistent with previous evidence demonstrating the benefits of multicomponent functional training interventions on physical fitness adaptations in athletic populations [33]. In addition, the effect of yoga on balance and joint kinematics has been noted in athletic populations, which further underscores the positive effects of yoga on improving balance and movement efficiency [16].

### 4.2. Physiological Adaptations

The review also brings attention to major physiological changes that occur due to Suryanamaskar and yoga-based approaches. Among studies that assessed cardiovascular and physiological outcomes, improvements in VO2 max, resting heart rate, blood pressure, and related indicators were generally reported. For example, Suwannakul et al. (2024) showed increases in VO2 max and decreases in body mass index and body fat percentage, which is related to enhanced cardiorespiratory efficiency [12]. In another study, Chawla et al. (2022) found decreases in heart rate and blood pressure after doing Suryanamaskar practice [27]. The results of this study align with the previous studies that indicate yoga practices have a beneficial effect on autonomic regulation, increase parasympathetic activity and optimize cardiovascular responses [9,10]. Further, these interventions showed a decrease in body composition parameters like BMI and waist circumference, which also suggest the metabolic benefits of these interventions [11]. The dynamic movement of Suryanamaskar coupled with controlled breathing probably enhances the use of oxygen and energy metabolism.

### 4.3. Psychological Outcomes

One of the most important findings of this review is that yoga and Suryanamaskar interventions are consistently shown to have positive effects on psychological well-being. Several studies indicated a decrease in stress, anxiety, and depression, as well as an increase in emotional intelligence, resilience and general well-being. In one study, Stec et al. (2023) found that students who practiced dynamic Suryanamaskar experienced substantial decreases in perceived stress and increases in emotional intelligence [21]. Likewise, Badve et al. (2025) found significant improvements in scores for psychological health parameters, such as decreased anxiety and increased quality of life in perimenopausal women [19]. This is congruent with neurophysiological evidence that yoga can modulate systems of response to stress and enhance emotional regulation via mechanisms that include the autonomic nervous system and neuroendocrine pathways [17,18]. Breathing, movement, and mindfulness combined in Suryanamakar are integral to the practice and could be the main factor in decreasing psychological tension and enhancing mental strength.

### 4.4. Combined and Multidimensional Effects

Several studies employing multidimensional yoga programmes that incorporated Suryanamaskar reported favourable outcomes across physical, physiological, psychological, and body-composition domains. For example, interventions combining Suryanamaskar with pranayama, mindfulness practices, or resistance training components were associated with improvements in body composition, stress reduction, physical fitness, and psychological well-being [11,19,31,32]. These findings suggest that yoga programmes incorporating multiple complementary components may provide broad health and performance benefits. However, direct comparisons between combined interventions and standalone Suryanamaskar interventions were not consistently performed across the included studies. Therefore, conclusions regarding the superiority of one intervention approach over another cannot currently be established. Overall, the findings support the concept of yoga as a multidimensional mind–body practice capable of influencing multiple health domains simultaneously [23,34].

### 4.5. Consistency and Strength of Evidence

Although the magnitude of effects varied across studies, most included investigations reported favorable outcomes following Suryanamaskar and yoga-based interventions incorporating Suryanamaskar. Positive trends were observed across physical fitness, physiological, psychological, and body-composition domains, with several studies reporting statistically significant improvements and moderate-to-large effect sizes, particularly for flexibility, muscular fitness, stress reduction, and psychological well-being. However, differences in study design, participant characteristics, intervention protocols, comparator conditions, and outcome measures contributed to variability in the observed effects and should be considered when interpreting the findings. Evidence from randomized and controlled studies generally provided greater confidence in the reported outcomes than evidence from quasi-experimental or self-controlled designs. Furthermore, the overall pattern of findings is broadly consistent with previous systematic reviews and meta-analyses reporting beneficial effects of yoga on physical and psychological health outcomes [35], thereby providing additional support for the potential value of Suryanamaskar-based interventions while highlighting the need for further high-quality research.

### 4.6. Mechanisms Underlying the Observed Effects

The underlying physiological and neuromuscular mechanisms constitute an important part in understanding the effectiveness of Suryanamaskar and yoga-based interventions. The specific progression of postures and the controlled breathing sequence result in a specific relationship between musculoskeletal activation and autonomic regulation. The movement between the postures increases the engagement of muscles, the joint mobility and circulation, controlled breathing improves oxygen delivery and autonomic balance [10]. This cross-modality stimulation is believed to help adapt the central and peripheral systems such as better neuromuscular coordination, improved cardiovascular efficiency and better metabolic regulation [7,23]. Furthermore, the consciousness of mindfulness practice can impact neural pathways linked to attention, emotional control, and stress control mechanisms, which can help with improving psychological functioning [18,34]. These effects seem to work together to offer a reasonable explanation for the concurrent gains across physical, physiological, and psychological areas.

### 4.7. Variability and Consistency Across Studies

Variation was observed across studies with respect to participant characteristics, study design, intervention duration, intervention composition, and outcome assessment methods. Differences between standalone Suryanamaskar protocols and broader yoga-based interventions incorporating Suryanamaskar, as well as variations in training frequency and intensity, likely contributed to differences in the magnitude of observed effects. Nevertheless, favorable outcomes were reported across a range of populations, including healthy adults, athletes, university students, older adults, and selected clinical groups. Although the overall pattern of findings suggests potential benefits across diverse settings, the generalizability of the results should be interpreted with caution because of methodological heterogeneity, differences in intervention protocols, and variability in study quality. Future research employing standardized interventions and outcome measures is needed to strengthen confidence in the consistency and applicability of these findings [17,22].

An important consideration when interpreting the present findings is that several included studies evaluated multidimensional yoga programmes in which Suryanamaskar was combined with other components such as pranayama, meditation, mindfulness practices, or resistance-training elements. Consequently, the observed benefits cannot always be attributed exclusively to Suryanamaskar, and the findings should be interpreted within the context of the broader intervention framework.

### 4.8. Limitations of the Study

The study has a few limitations. Although this review was conducted comprehensively and systematically, there are some limitations which need to be recognized. Firstly, there was large variability in study participants, intervention duration, frequency and outcome measures across the studies included, making a meta-analysis impossible. Secondly, while a great number of studies used randomized controlled designs, a few used a quasi-experimental or self-controlled design, which may have caused some selection bias and reduced internal validity. Third, some studies did not report an effect size or statistical parameters, leading to some difficulties in direct comparison of the size of the outcomes. Fourth, most of the studies were performed in India, and there is a possibility that its results may not be generalizable across cultural or geographic contexts. Fifth, the results could be affected by the different intervention protocols (standalone Suryanamaskar vs. combined yoga programs). Sixth, there were some studies with relatively small sample sizes, which can decrease statistical power and enhance the likelihood of a Type II error. Lastly, the use of published, English-language, peer-reviewed studies may have resulted in publication bias, which may have eliminated relevant work from grey or non-English literature. A further limitation of this review is that a formal certainty-of-evidence assessment using the Grading of Recommendations Assessment, Development and Evaluation (GRADE) framework was not conducted. Given the substantial heterogeneity in study designs, participant characteristics, intervention protocols, comparator conditions, and outcome measures, quantitative synthesis and standardized certainty assessment across outcome domains were not considered feasible. Consequently, the overall confidence in the available evidence should be interpreted with caution, and future reviews incorporating a larger number of methodologically comparable studies may benefit from applying formal certainty-of-evidence frameworks.

### 4.9. Practical Implications

The conclusions of this review have major implications for various groups of stakeholders such as teachers, coaches, health care professionals, policymakers and the public. Suryanamaskar can be effectively incorporated into the school and university curriculum systematically as a space-saving, low-cost and comprehensive exercise training. It can be used as an element in training routines for coaches and sports practitioners to increase their flexibility, balance, endurance, and recovery, ultimately boosting their athletic performance. Suryanamaskar can be used as a non-pharmacologic therapy for stress, obesity, and lifestyle-related diseases by healthcare professionals and rehabilitation specialists, as part of preventive healthcare programs. The current evidence suggests that Suryanamaskar may represent a practical and accessible movement-based intervention that can be incorporated into educational, community, recreational, and health-promotion settings. However, given the methodological limitations and heterogeneity of the available literature, policy-level recommendations should await confirmation from larger and more rigorous controlled trials. Overall, the multifaceted advantages discussed in this review highlight that Suryanamaskar can be implemented as an evidence-based, scalable, and inclusive intervention across the performance, health, and wellness domains.

## 5. Conclusions

The findings of this systematic review suggest that Suryanamaskar and yoga-based interventions incorporating Suryanamaskar may contribute to improvements in physical fitness, physiological function, psychological well-being, and body composition across diverse populations. Positive effects were most consistently observed in muscular strength, endurance, flexibility, balance, cardiovascular fitness, and psychological outcomes such as stress, anxiety, and depressive symptoms. The combination of dynamic movement, controlled breathing, and attentional focus may provide a holistic approach capable of influencing multiple health-related domains simultaneously. However, these findings should be interpreted with caution due to heterogeneity in study designs, participant characteristics, intervention protocols, and outcome measures, as well as the difficulty of isolating the specific effects of Suryanamaskar from broader yoga-based interventions. Nevertheless, the available evidence suggests that Suryanamaskar may represent a practical and accessible component of physical education, athletic training, and health-promotion programs. Although favourable outcomes were reported across multiple domains, several interventions incorporated additional yoga practices or exercise components alongside Suryanamaskar. Therefore, the independent contribution of Suryanamaskar to the observed effects cannot always be clearly distinguished. Future research should employ rigorous randomized controlled designs, larger and more diverse samples, standardized intervention protocols, and consistent outcome reporting to strengthen the evidence base and improve the generalizability of findings.

## Figures and Tables

**Table 1:** Inclusion and Exclusion Criteria.

Component	Inclusion Criteria	Exclusion Criteria
P (Population)	Human participants of any age group (adolescents, adults, elderly), both sexes; includes healthy individuals, athletes, and clinical populations (e.g., obesity, poor sleep quality, perimenopausal women, intellectual disability)	Animal studies; participants with severe uncontrolled medical conditions; studies focusing exclusively on rehabilitation of acute injuries or surgical populations
I (Intervention)	Studies involving Suryanamaskar or yoga interventions incorporating Suryanamaskar (alone or combined with asanas, pranayama, meditation, resistance training, or flexibility protocols)	Studies not involving Suryanamaskar or yoga-based interventions; purely meditation-only or pranayama-only interventions without physical components
C (Comparator)	Studies with control groups (active, passive, or no intervention), alternative exercise (e.g., stretching, walking, cycling), or pre–post comparisons (self-controlled designs)	Studies lacking any form of comparison (e.g., purely descriptive studies without pre–post data); theoretical or opinion-based papers
O (Outcomes)	At least one measurable outcome related to: physical fitness (strength, endurance, flexibility, balance), physiological parameters (VO2 max, HRV, BP), psychological outcomes (stress, anxiety, depression, well-being), cognitive performance, or body composition	Studies not reporting quantitative outcomes; studies reporting only qualitative outcomes without measurable data
Study Design	Experimental, quasi-experimental, randomized controlled trials, crossover designs, and intervention-based pre–post studies	Case studies, case reports, reviews, editorials, conference abstracts without full data, and non-peer-reviewed articles
Language & Publication	Studies published in English in peer-reviewed journals	Non-English studies; unpublished theses, dissertations, or grey literature (unless peer-reviewed)
Time Frame	No strict time restriction; studies included up to the latest available (reflecting contemporary evidence up to 2025)	Studies published outside accessible time frame or lacking full-text availability

**Table 2:** Characteristics of the Included Studies Evaluating the Effects of Surya Namaskar and Surya Namaskar-Based Yoga Interventions (N = 14).

Study (Author & Year)	Country	Design	Sample/Population	Classification	Intervention (SN/Yoga Protocol)	Comparator/Control	Duration & Frequency	Outcome Variables	Key Findings
Bhutkar et al. (2011) [13]	India	Sequential self-control study	79 medical undergraduate students (49 males, 30 females), aged 17.5–20 years; excluded those with prior sports training, yoga practice, or medical illness	Standalone Suryanamaskar	Structured practice of Suryanamaskar involving 10-step cyclical movements synchronized with breathing; cycles increased from 6 to 24 progressively	No separate control group; participants served as their own control (pre–post comparison)	24 weeks; 6 days/week; 1 h/session (5:00–6:00 PM)	Muscle Strength (bench press, shoulder press, back & leg dynamometry); General Endurance (push-ups, sit-ups); Body Composition (BMI, % body fat, lean body mass); Perceived Exertion (Borg scale)	Significant improvements in upper and lower body strength (*p* < 0.001–0.0002); improved endurance (push-ups, sit-ups; *p* < 0.001); reduced BMI and body weight (*p* < 0.0001); body fat % decreased significantly in females only; lean body mass increased (significant in females); females showed greater overall improvement; RPE: males “fairly light” (~50% VO2 max), females “somewhat hard” (~75% VO2 max)
Mangaonkar & Puntambekar (2018) [15]	India	Comparative experimental study	30 physiotherapy students (aged 19–25 years) with restricted hamstring flexibility (knee extension range < 50°)	Standalone Suryanamaskar	Suryanamaskar: 8 cycles per session; each posture held for 5 s (~60-s cycle per round)	Dynamic stretching protocol: 1 set of 8 exercises (straight leg strides, inchworm, walking diagonal lunges, carioca, low lateral shifts, single-step Romanian deadlift, backward run, high knee pulls); 1 min per exercise (30 s per leg)	4 weeks; ~15 min/session (including 5 min warm-up and 2 min cooldown)	Hamstring flexibility assessed via Active Knee Extension (AKE) test and Back Saver Sit-and-Reach (BSSR) test	Both interventions significantly improved bilateral hamstring flexibility (*p* < 0.0001); Suryanamaskar demonstrated significantly greater improvement compared to dynamic stretching (*p* < 0.0001).
Chawla et al. (2022) [27]	India	Quasi-experimental study	30 young adults aged 18–40 years with poor sleep quality (PSQI > 5)	Standalone Suryanamaskar	Suryanamaskar: 12-posture sequence with controlled breathing; 3 sessions/week; 40–45 min/session; progressive increase in sets	Control group: 20 min daily walking program	8 weeks; experimental: 3 sessions/week; control: daily walking	Sleep quality (Pittsburgh Sleep Quality Index—PSQI); Psychological well-being (PGWBI); Resting heart rate; Blood pressure; Body Mass Index (BMI)	Significant improvements in sleep quality, psychological well-being, resting heart rate, systolic blood pressure, and BMI in the intervention group; control group showed improvement only in general well-being; significant between-group differences favored the Suryanamaskar group for sleep quality, psychological well-being, and heart rate.
Stec et al. (2023) [21]	India	Solomon’s four-group design	Indian male teenage school students; four-group structure with subgroup sizes up to n ≈ 27 for post-test measurements	Standalone Dynamic Suryanamaskar	Dynamic Suryanamaskar (DSN): 12-posture Rishikesh series performed with rapid transitions (7.5–8 s/round); protocol included ~35 min DSN, 10 min flexibility exercises (first 4 weeks), and 5 min relaxation	Control groups: stretching exercises and jogging (~20 min)	12 weeks; 6 days/week	Psychological outcomes: Perceived Stress Scale (PSS); Emotional Intelligence (EQ questionnaire for Indian population)	Significant reduction in perceived stress in DSN groups (Groups I & III) compared to control groups (Groups II & IV), where stress increased (likely due to examination pressure); significant improvement in emotional intelligence in DSN groups, with stronger pre–post gains compared to controls and higher post-test trends; overall, DSN demonstrated superior effectiveness over jogging and stretching in enhancing psychological well-being and reducing negative emotional states
Raja (2023) [28]	India	Experimental two-group design	30 older men (50–60 years; mean age 56.3 ± 1.3) residing in an old-age home; randomly divided into experimental (n = 15) and control (n = 15) groups	Standalone Suryanamaskar	Suryanamaskar: 12-posture sequence including Pranamasana, Hasta Uttanasana, Padahastasana, Ashwa Sanchalanasana, Ashtanga Namaskara, Bhujangasana, and Parvatasana	Control group: routine daily activities without structured training	12 weeks; 6 days/week; twice daily sessions (morning and evening)	Psychological: Depression (Hamilton Depression Scale); Physical: Trunk flexibility (sit-and-reach test)	Significant reduction in depression levels and significant improvement in trunk flexibility in the Suryanamaskar group compared to the control group.
Ethiraj et al. (2024) [14]	India	Experimental two-group pretest–post-test design	30 male college students (18–20 years), randomly assigned to treatment (n = 15) and control (n = 15) groups	Standalone Suryanamaskar	Surya Namaskar practice program performed 5 days/week	Control group: no intervention; maintained regular daily activities	6 weeks; 5 sessions/week	Flexibility: Back flexibility (sit-and-reach test); Lumbar flexion	Significant improvement in back flexibility (t = 17.75, *p* < 0.001) and lumbar flexion (t = 3.86, *p* = 0.002) in the intervention group; no significant changes in control group; findings support the effectiveness of Suryanamaskar in enhancing spinal flexibility and kinanthropometric parameters in young adults
Devi et al. (2024) [11]	India	Randomized controlled pre–post study design	60 participants (male and female), aged 18–30 years, diagnosed with Grade 1 obesity (WHO criteria); recruited from naturopathy and yoga centers in Moodabidri	Yoga Program with Explicit Suryanamaskar	Suryanamaskara (15 rounds, ~30 min) combined with heating pranayama (Kapalabhati, Bhastrika, Surya Anuloma Viloma; ~3 min each); total session ~45 min	Control group: no intervention; maintained regular daily activities	4 weeks; 5 sessions/week	Body composition: Body Mass Index (BMI), Waist Circumference (WC), Skinfold Thickness (abdominal, triceps, subscapular)	Significant reductions in BMI, waist circumference, and skinfold thickness in the intervention group after 4 weeks; the control group showed no improvement or slight increases in some parameters, indicating effectiveness of the combined yoga protocol in managing obesity-related parameters
Suwannakul et al., (2024) [12]	Thailand	Single-blinded randomized controlled trial	44 overweight/obese female university students (19–22 years); 43 completed (SN group n = 21, control n = 22)	Standalone Suryanamaskar	Surya Namaskar yoga training program	Control group: sedentary (no participation in structured exercise)	8 weeks; 3 sessions/week	Psychological: Perceived stress (T-PSS-10); Body composition: BMI, Waist–Hip Ratio (WHR), Body fat %; Physiological: VO2 max; Strength: Hand grip, leg strength; Flexibility: Forward-back flexibility	Within the SN group: significant improvements (*p* < 0.05) in flexibility, hand grip strength, leg strength, and VO2 max; significant reductions in perceived stress and BMI; Between groups: significant differences favoring SN group in flexibility (*p* = 0.015) and stress (*p* = 0.009); the control group showed no significant changes.
Dubey & Choudhary (2024) [29]	India	Experimental two-group pretest–post-test design	30 healthy female college students (mean age ≈ 20.8–20.9 years), randomly assigned to experimental (n = 15) and control (n = 15) groups	Standalone Suryanamaskar	Structured Surya Namaskar regimen: 5–12 min warm-up, 12–15 min Suryanamaskar (12 postures held ~6 s each; starting with 4 cycles, progressively increased every 2 weeks), 5–7 min cool-down and prayer	Control group: routine activities, no intervention	6 weeks	Flexibility: Back flexibility; Lumbar flexion	Experimental group showed significant improvements in back flexibility (3.20 ± 0.95 cm; t = 16.45, *p* = 0.000) and lumbar flexion (1.50 ± 0.75 cm; t = 3.95, *p* = 0.001); control group showed no significant changes; findings support effectiveness of Suryanamaskar in enhancing musculoskeletal flexibility in female college students.
Choudhary et al. (2025) [30]	India	12-week quasi-experimental pre–post design	28 college athletes: Yoga Group (YG, n = 14, handball players; mean age 19.8 ± 1.05 years) and Non-Yoga Group (NYG, n = 14, basketball players; mean age 20.3 ± 1.06 years);	Yoga Program with Explicit Suryanamaskar	Supervised yoga intervention (including Suryanamaskar elements): 60 min/session, twice weekly (warm-up, asana practice, cool-down), conducted prior to other physical activity	Non-Yoga Group: regular sport-specific training (static stretching, resistance training, aerobic conditioning); no additional intervention	12 weeks; Yoga Group: 2 sessions/week (60 min/session)	Flexibility (Sit-and-Reach, Shoulder Flexibility test); Balance (Stork Stand test); Joint Kinematics (Right Forward Lunge, Downward Dog, Chair Pose angles)	Yoga Group showed significant improvements: Sit-and-Reach (+2.1 inches, *p* = 0.009), Shoulder Flexibility (+1.0 inch, *p* = 0.025), Stork Stand (+5.1 s, *p* = 0.018), and favorable joint angle adaptations; Non-Yoga Group showed negligible or regressive changes in most variables; significant between-group differences observed in Sit-and-Reach (*p* = 0.04) and Stork Stand (*p* = 0.04), indicating superior effectiveness of yoga intervention
Choudhary et al. (2025) [31]	India	Randomized controlled trial	34 male intermediate-level tennis athletes (18–23 years), Eclipse category	Yoga Program with Explicit Suryanamaskar	Combined yoga (including Suryanamaskar-related movement patterns and balance-centric asanas such as Tree Pose and Warrior III) + elastic-band resistance training with progressive overload, unilateral loading, and resisted sprint integration (weeks 9–12)	Control group: regular tennis training only	12 weeks; supervised sessions; ≥80% adherence	Functional fitness: Chair Stand (lower body endurance), Arm Curl (upper body endurance), Sit-and-Reach (flexibility), Single-Leg Stance (balance), Timed Up-and-Go (agility)	Significant improvements in experimental group compared to control in muscular endurance (upper & lower body), flexibility, agility, and balance; large effect sizes particularly in arm curl, sit-and-reach, and agility measures; findings indicate strong neuromuscular and functional performance adaptations relevant to tennis.
Badve et al. (2025) [19]	India	Randomized controlled trial	100 perimenopausal women (Yoga group n = 50; Control group n = 50); mean age: 46.5 ± 4.2 (Yoga), 47.1 ± 3.9 (Control)	Yoga Program with Explicit Suryanamaskar	Structured Hatha Yoga program incorporating asanas, pranayama, meditation, and relaxation (including elements related to Suryanamaskar practice context): 75 min/session, 5 days/week	Control group: no intervention	12 weeks; 5 sessions/week; 75 min/session	Psychological: HDRS (depression), HARS (anxiety), PSQI (sleep), Greene Climacteric Scale, PSS (stress), WHOQOL-BREF (quality of life), RSES (self-esteem), BFI (neuroticism); Additional: hormonal and anthropometric measures	Significant improvements in yoga group compared to control: reduced depression (−6.3), anxiety (−6.8), stress (−7.2), poor sleep (−5.4), climacteric symptoms (−9.4), and neuroticism (−9.6); increased self-esteem (+5.6) and quality of life (+14.4); control group showed minimal or no changes
Vijayalakshmi and Saroja (2025) [32]	India	Two-group randomized controlled experimental (pre–post) design	30 competitive female basketball players (19–24 years), divided into experimental group (n = 15) and control group (n = 15)	Yoga Program with Explicit Suryanamaskar	Structured yoga intervention (including elements related to Suryanamaskar): ~45 min/session comprising 10 min pranayama, 25 min asanas, and 10 min mindfulness/relaxation; progressively advanced protocol (breathing, postures, visualization, muscle relaxation)	Control group: routine basketball training only	8 weeks; 3 sessions/week (24 sessions total)	Cognitive: d_2_ Test of Attention; Psychological: CD-RISC-10 (resilience), PSS-10 (stress); Functional: vertical jump, sprint, agility	Significant improvements in experimental group compared to control: enhanced cognitive focus (*p* < 0.001), psychological resilience (*p* = 0.004), reduced perceived stress (*p* = 0.002), and improved agility (*p* = 0.011); substantial gains in vertical jump performance; overall improvement in psychophysiological readiness and functional efficiency
Udiyapuram et al. (2025) [16]	India	Randomized controlled trial (RCT)	41 male basketball players (≈18–24 years); Yoga Asanas Group (n = 21) and Control Group (n = 20)	Yoga Program with Explicit Suryanamaskar	Yogic asanas program including Surya Namaskar, Utkatasana, Virabhadrasana, Tadasana, Trikonasana, Vrksasana, Bhujangasana, and Savasana.	Control group: continued regular basketball training without yoga	12 weeks; 3 sessions/week; 60 min/session	Physical fitness: Cardiorespiratory endurance (CVE), Muscular strength (MS), Muscular endurance (ME), Flexibility (FLE)	Significant improvements in yoga group: CVE (+8.01%), MS (+8.50%), ME (+11.41%), and FLE (+9.80%) (all *p* < 0.001); control group showed no significant changes; findings support yoga as an effective complementary training modality for enhancing physical fitness in basketball players.

Note: AKE—Active Knee Extension; BSSR—Back Saver Sit and Reach; BMI—Body Mass Index; BP—Blood Pressure; CVE—Cardiovascular Endurance; DBP—Diastolic Blood Pressure; DSN—Dynamic Suryanamaskar; EQ—Emotional Intelligence; FLE—Flexibility; HR—Heart Rate; HRV—Heart Rate Variability; LF—Lumbar Flexion; MVV—Maximum Voluntary Ventilation; PEFR—Peak Expiratory Flow Rate; PSS—Perceived Stress Scale; PSQI—Pittsburgh Sleep Quality Index; PGWBI—Psychological General Well-Being Index; RCT—Randomized Controlled Trial; SBP—Systolic Blood Pressure; SN—Suryanamaskar; VO2 max—Maximum Oxygen Consumption.

**Table 3:** RoB 2 assessment for randomized studies.

No.	Study	Outcome Assessed	D1 Bias Arising from the Randomization Process	D2 Bias Due to Deviations from Intended Interventions	D3 Bias Due to Missing Outcome Data	D4 Bias in Measurement of the Outcome	D5 Bias in Selection of the Reported Result	Overall RoB 2 Judgement
1	Stec et al. (2023) [21]	Perceived stress/emotional intelligence	Some concerns	Some concerns	Some concerns	Some concerns	Some concerns	Some concerns
2	Ethiraj et al. (2024) [14]	Back flexibility/lumbar flexion	Some concerns	Some concerns	Low/Some concerns	Some concerns	Some concerns	Some concerns
3	Devi et al. (2024) [11]	BMI/body composition/obesity-related outcomes	Some concerns	Some concerns	Some concerns	Some concerns	Some concerns	Some concerns
4	Suwannakul et al. (2024/2025) [12]	Perceived stress/anthropometric parameters/physical fitness	Some concerns	Some concerns	Some concerns	Some concerns	Some concerns	Some concerns
5	Dubey and Choudhary (2024) [29]	Back flexibility/lumbar flexion	Some concerns	Some concerns	Low/Some concerns	Some concerns	Some concerns	Some concerns
6	Choudhary et al. (2025) [31]	Functional fitness/strength/flexibility/mobility/balance	Some concerns	Some concerns	Some concerns	Some concerns	Some concerns	Some concerns
7	Badve et al. (2025) [19]	Depression/anxiety/stress/sleep/QoL/self-esteem	Some concerns	Some concerns	Some concerns	High	Some concerns	High
8	Udiyapuram et al. (2025) [16]	Endurance/strength/flexibility	Some concerns	Some concerns	Low/Some concerns	Some concerns	Some concerns	Some concerns
9	Vijayalakshmi and Saroja (2025) [32]	Cognitive focus/resilience/perceived stress/functional performance	Some concerns	Some concerns	Some concerns	Some concerns	Some concerns	Some concerns

Note: RoB 2 = Cochrane Risk of Bias 2 tool; D1 = Bias arising from the randomization process; D2 = Bias due to deviations from intended interventions; D3 = Bias due to missing outcome data; D4 = Bias in measurement of the outcome; D5 = Bias in selection of the reported result; BMI = Body Mass Index; QoL = Quality of Life. Domain-level and overall judgements were classified according to the Cochrane RoB 2 guidance as ‘Low risk’, ‘Some concerns’, or ‘High risk’ of bias.

**Table 4:** ROBINS-I assessment for non-randomized studies.

No.	Study	Outcome Assessed	D1 Bias Due to Confounding	D2 Bias in Selection of Participants into the Study	D3 Bias in Classification of Interventions	D4 Bias Due to Deviations from Intended Interventions	D5 Bias Due to Missing Data	D6 Bias in Measurement of Outcomes	D7 Bias in Selection of the Reported Result	Overall ROBINS-I Judgement
1	Bhutkar et al. (2011) [13]	Muscle strength, muscular endurance, body composition, BMI	Serious	Moderate/Serious	Low/Moderate	Serious	Moderate	Moderate	Moderate	Serious
2	Mangaonkar & Puntambekar (2018) [15]	Hamstring flexibility/sit-and-reach/active knee extension	Serious	Moderate	Moderate	Serious	Moderate	Moderate	Moderate	Serious
3	Raja (2023) [28]	Depression/trunk flexibility	Serious	Moderate	Moderate	Serious	Moderate	Moderate/Serious	Moderate	Serious
4	Chawla et al. (2022) [27]	Sleep quality/general well-being/HR/BP/BMI	Serious	Moderate	Moderate	Serious	Moderate	Serious	Moderate	Serious
5	Choudhary et al. (2025) [30]	Flexibility/balance/kinematics	Serious	Moderate	Moderate	Serious	Moderate	Moderate	Moderate	Serious

Note: ROBINS-I = Risk Of Bias In Non-randomized Studies of Interventions; D1 = Bias due to confounding; D2 = Bias in selection of participants into the study; D3 = Bias in classification of interventions; D4 = Bias due to deviations from intended interventions; D5 = Bias due to missing data; D6 = Bias in measurement of outcomes; D7 = Bias in selection of the reported result; HR = Heart Rate; BP = Blood Pressure; BMI = Body Mass Index. Domain-level and overall judgements were classified according to the ROBINS-I guidance as ‘Low’, ‘Moderate’, ‘Serious’ risk of bias.

**Table 5:** Summary of Effects of Suryanamaskar and Yoga-Based Interventions on Physical, Physiological, and Psychological Outcomes.

Author, Year	Condition	Outcome Measure	Pre-Mean (SD)	Post Mean (SD)	Mean Difference	Statistical Significance	Effect Magnitude	Direction
Bhutkar et al. (2011) [13]	Suryanamaskar (Males & Females)	Bench Press; Sit-ups; BMI; Body Fat %	Males: BP 29.49 ± 9.70; Sit-ups 24.92 ± 10.41; BMI 21.43 ± 3.91; BF 18.84 ± 6.29; Females: BP 10.5 ± 4.42; Sit-ups 13.16 ± 7.75; BMI 22.41 ± 3.88; BF 27.68 ± 5.46	Males: BP 36.12 ± 9.09; Sit-ups 29.84 ± 12.64; BMI 20.87 ± 3.61; BF 18.42 ± 5.56; Females: BP 13.16 ± 4.44; Sit-ups 19.23 ± 8.25; BMI 21.76 ± 3.64; BF 25.76 ± 4.72	Males: BP +6.63; Sit-ups +4.92; BMI −0.56; BF −0.42; Females: BP +2.66; Sit-ups +6.07; BMI −0.65; BF −1.92	Significant for all except male body fat (*p* = 0.06); others *p* ≤ 0.0005	Males: BP +22.49%; Sit-ups +19.74%; BMI −2.44%; BF −2.25%; Females: BP +25.33%; Sit-ups +46.13%; BMI −3.29%; BF −6.95%	↑ Strength & endurance; ↓ BMI & body fat (greater improvements in females)
Mangaonkar & Puntambekar (2018) [15]	Hamstring Flexibility	AKE Right & Left; Back Saver Sit & Reach Right & Left	SN: ~34–35° (AKE), ~12–13 cm (BSSR); DS: ~34–35° (AKE), ~12–13 cm (BSSR)	SN: ~47° (AKE), ~20–21 cm (BSSR); DS: ~43–44° (AKE), ~17–18 cm (BSSR)	SN: +12.47° to +12.80° (AKE), +7.53 to +8.20 cm (BSSR); DS: +8.47° (AKE), +5.27 to +5.47 cm (BSSR)	*p* < 0.0001 (within-group and between-group)	Not reported (described as “extremely significant”)	SN > Dynamic Stretching
Chawla et al. (2022) [27]	Experimental Group (Suryanamaskar)	PSQI; PGWBI; Heart Rate; Systolic BP; Diastolic BP; BMI	PSQI 13.93 ± 2.66; PGWBI 43.20 ± 12.42; HR 90.73 ± 9.81; SBP 121.20 ± 4.14; DBP 79.47 ± 4.34; BMI 23.34 ± 3.11	PSQI 9.53 ± 1.68; PGWBI 78.33 ± 14.76; HR 77.07 ± 5.48; SBP 116.67 ± 2.99; DBP 78.73 ± 2.79; BMI 23.01 ± 3.17	PSQI −4.40; PGWBI +35.13; HR −13.66; SBP −4.53; DBP −0.74; BMI −0.33	Significant for PSQI, PGWBI, HR, SBP, BMI (*p* ≤ 0.019); Not significant for DBP (*p* = 0.275)	Significant for PSQI, PGWBI, HR, SBP, BMI; Not significant for DBP	Improved sleep & wellbeing; ↓ HR, ↓ BP, ↓ BMI
Stec et al. (2023) [21]	DSN vs. Control (Groups I–IV)	Perceived Stress Scale (PSS); Emotional Intelligence (EQ)	PSS (G I): 19.77 ± 4.41; PSS (G II): 18.04 ± 4.75; EQ (G I): 159.92 ± 31.78; EQ (G II): 155.88 ± 28.71	PSS (G I): 17.73 ± 2.79; PSS (G II): 20.28 ± 4.24; EQ (G I): 171.08 ± 29.18; EQ (G II): 152.00 ± 26.39	PSS: −2.04 (G I), +2.24 (G II); EQ: +11.16 (G I), −3.88 (G II)	Significant reduction in stress (F (1,48) = 19.5, *p* < 0.001); significant increase in EQ (F (1,48) = 11.133, *p* = 0.002); post-test comparison: PSS *p* = 0.011 (significant), EQ *p* = 0.081 (not significant)	Partial η^2^: 0.198–0.436 (within groups), 0.188–0.289 (between groups)	↓ Stress; ↑ Emotional Intelligence (DSN superior to control)
Raja (2023) [28]	Suryanamaskar Practice Group	Depression; Trunk Flexibility	Depression: 19.53 ± 0.83; Flexibility: 3.98 ± 0.20	Depression: 14.80 ± 0.94; Flexibility: 4.25 ± 0.19	Depression: −4.73; Flexibility: +0.27	Depression: t = 4.58, *p* < 0.05; Flexibility: t = 3.72, *p* < 0.05 (statistically significant)	Depression: t = 4.58, F = 165.31; Flexibility: t = 3.72, F = 11.03	↓ Depression; ↑ Flexibility
Ethiraj et al. (2024) [14]	Treatment (Suryanamaskar) vs. Control	Back Flexibility (BF); Lumbar Flexion (LF)	Treatment: BF 30.47 ± 1.13; LF 6.80 ± 1.42; Control: BF 30.40 ± 0.83; LF 6.27 ± 0.88	Treatment: BF 33.47 ± 1.12; LF 8.20 ± 0.86; Control: BF 30.27 ± 0.88; LF 6.33 ± 0.96	Treatment: BF +3.00; LF +1.40; Control: BF −0.13; LF +0.06	Significant in treatment (BF *p* < 0.001; LF *p* = 0.002); Not significant in control (*p* > 0.05)	BF t = 17.75; LF t = 3.86	↑ Flexibility in treatment; no meaningful change in control
Devi et al. (2024) [11]	Experimental (SN + Pranayama) vs. Control	Weight; BMI; Waist Circumference; Abdominal Circumference; Triceps Circumference	Exp: Weight 79.93 ± 7.88; BMI 32.93 ± 1.09; WC 110.03 ± 5.79; Abd 36.07 ± 3.54; Tri 33.73 ± 2.91; Ctrl: Weight 82.6 ± 5.54; BMI 33.26 ± 0.98; WC 116.87 ± 9.25; Abd 36.6 ± 1.91; Tri 33.73 ± 2.45	Exp: Weight 77.43 ± 7.75; BMI 31.92 ± 1.19; WC 108.2 ± 5.6; Abd 33.80 ± 3.58; Tri 32.07 ± 2.9; Ctrl: Weight 83.17 ± 5.47; BMI 33.50 ± 0.96; WC 117.8 ± 8.86; Abd 36.8 ± 1.79; Tri 34.47 ± 2.86	Exp: Weight −2.50; BMI −1.01; WC −1.83; Abd −2.27; Tri −1.66; Ctrl: minimal or adverse changes	Significant improvements in the experimental group (*p* ≤ 0.05); no significant improvement in the control	0.303–0.78 (moderate to large)	↓ Body weight, BMI, and circumferences in the experimental group; no improvement in the control.
Suwannakul et al., (2024) [12]	SN Yoga Group vs. Control	Perceived Stress (PSS); BMI; WHR; Body Fat %; Flexibility; Hand Grip Strength; Leg Strength; VO2 max	SN: PSS 17.50 ± 3.50; BMI 27.50 ± 2.50; WHR 0.85 ± 0.05; BF% 33.50 ± 3.00; Flex 15.00 ± 2.50; Grip 24.50 ± 2.00; Leg 74.00 ± 7.50; VO2 max 36.00 ± 3.50; Control: similar baseline values	SN: PSS 13.50 ± 3.00; BMI 26.00 ± 2.50; WHR 0.80 ± 0.05; BF% 32.50 ± 3.00; Flex 18.00 ± 2.00; Grip 26.50 ± 2.00; Leg 79.00 ± 7.00; VO2 max 38.00 ± 3.00; Control: minimal/no change	SN: PSS −4.00; BMI −1.50; WHR −0.05; BF% −1.00; Flex +3.00; Grip +2.00; Leg +5.00; VO2 max +2.00; Control: negligible changes	Significant within-group improvements in SN group (*p* < 0.05); between-group significant for PSS (*p* = 0.009) and flexibility (*p* = 0.015); others not significant between groups	Not reported (interpreted via mean change; study noted as underpowered for effect size detection)	↓ Stress, BMI, WHR, body fat; ↑ flexibility, strength, and VO2 max. Significant between-group differences favored the Suryanamaskar group for stress and flexibility.
Dubey & Choudhary (2024) [29]	Experimental (Suryanamaskar) vs. Control	Back Flexibility; Lumbar Flexion	Exp: BF 31.30 ± 1.05; LF 7.00 ± 1.25; Ctrl: BF 31.10 ± 0.90; LF 6.50 ± 0.80	Exp: BF 34.30 ± 1.00; LF 8.50 ± 0.90; Ctrl: BF 30.95 ± 0.85; LF 6.55 ± 0.88	Exp: BF +3.00; LF +1.50; Ctrl: BF −0.15; LF +0.05	Significant in experimental group (BF *p* = 0.000; LF *p* = 0.001); not significant in control (*p* > 0.05)	Exp: BF t = 16.45; LF t = 3.95; Ctrl: BF t = 1.32; LF t = 0.18	↑ Flexibility in experimental group; no meaningful change in control
Choudhary et al. (2025) [30]	Yoga Group (YG) vs. Non-Yoga Group (NYG)	Sit-and-Reach; Shoulder Flexibility; Stork Stand; Joint Angles (RFL, Downward Dog, Chair Pose)	YG: SR 21.6 ± 3.7; SF −0.2 ± 2.9; SS 13.4 ± 6.9; NYG: SR 21.2 ± 2.8; SF −1.0 ± 3.7; SS 21.9 ± 8.6	YG: SR 23.7 ± 2.4; SF 0.8 ± 2.7; SS 18.5 ± 7.8; NYG: SR 20.7 ± 3.4; SF −1.9 ± 2.8; SS 18.0 ± 9.3	YG: SR +2.1; SF +1.0; SS +5.1; NYG: SR −0.5; SF −0.9; SS −3.9; Joint angles (YG): significant improvements (e.g., hip +11.4°, knee +4.2°); NYG: regressive changes (e.g., hip −10.2°, knee −10.7°)	Significant improvements in YG (*p* = 0.008–0.039); no significant improvements or regressions in NYG (*p* > 0.05 for main outcomes; some negative significant changes in joint angles)	Reported as small–large (study-defined; not standardized)	↑ Flexibility, balance, and joint mechanics in YG; ↓ or regressive changes in NYG
Choudhary et al. (2025) [31]	12-week Yoga + Elastic-Band Training	Arm Curl; Chair Stand; Sit-and-Reach; Single-Leg Stance; Timed Up-and-Go	Arm Curl 15.2 ± 3.6; Chair Stand 14.3 ± 3.9; Sit-and-Reach 1.5 ± 2.7; Single-Leg Stance 10.4 ± 3.9; TUG 8.9 ± 0.7	Arm Curl 19.3 ± 3.8; Chair Stand 16.2 ± 4.1; Sit-and-Reach 7.3 ± 4.0; Single-Leg Stance 12.4 ± 5.1; TUG 7.9 ± 0.6	Arm Curl +4.1; Chair Stand +1.9; Sit-and-Reach +5.8; Single-Leg Stance +2.0; TUG −1.0	Significant improvements across all outcomes (*p* < 0.001)	Arm Curl d = 1.16; Chair Stand d = 0.61; Sit-and-Reach d = 1.35; Single-Leg Stance d = 0.25 (small); TUG d = −1.53	↑ Strength, flexibility, balance; ↓ time in agility (improvement)
Badve et al. (2025) [19]	Perimenopause (Yoga Intervention)	Depression (HDRS); Anxiety (HARS); Sleep (PSQI); Climacteric Symptoms; Stress (PSS); Quality of Life (WHOQOL); Self-Esteem; Neuroticism	HDRS 18.6 ± 4.2; HARS 22.4 ± 5.1; PSQI 12.8 ± 3.2; GCS 28.6 ± 6.4; PSS 25.4 ± 5.8; QoL 58.2 ± 12.4; RSES 16.8 ± 4.2; Neuroticism 62.4 ± 8.6	HDRS 12.3 ± 3.8; HARS 15.6 ± 4.2; PSQI 7.4 ± 2.6; GCS 19.2 ± 5.1; PSS 18.2 ± 4.6; QoL 72.6 ± 11.2; RSES 22.4 ± 3.8; Neuroticism 52.8 ± 7.4	HDRS −6.3; HARS −6.8; PSQI −5.4; GCS −9.4; PSS −7.2; QoL +14.4; RSES +5.6; Neuroticity −9.6	Significant across all outcomes (*p* ≤ 0.001)	d = 0.75–0.86 (moderate to large effects)	↓ Depression, anxiety, stress, neuroticism, symptoms; ↑ QoL and self-esteem
C & S (2025) [32]	Experimental (Yoga + Routine Training) vs. Control	Cognitive Focus (d2 TP); Resilience; Perceived Stress (PSS); Vertical Jump; 20 m Sprint; Agility	Exp: d2 712.4 ± 64.1; Resilience 61.3 ± 4.6; PSS 21.7 ± 5.1; VJ 27.5 ± 4.0; Sprint 3.91 ± 0.21; Agility 10.92 ± 0.54; Ctrl: similar baseline values	Exp: d2 652.2 ± 56.3; Resilience 68.8 ± 4.2; PSS 15.4 ± 4.7; VJ 31.8 ± 4.3; Sprint 3.78 ± 0.18; Agility 10.26 ± 0.50; Ctrl: minimal improvements	Exp: d2 −60.2; Resilience +7.5; PSS −6.3; VJ +4.3; Sprint −0.13; Agility −0.66; Ctrl: small/negligible changes	Significant improvements in experimental group (*p* < 0.001– 0.003); mostly non-significant in control (except small VJ improvement *p* = 0.048)	Exp: d = 0.81–1.84 (large effects); Ctrl: d = 0.16–0.42 (small/negligible)	Significant improvements were reported in cognitive focus, resilience, perceived stress, vertical jump, sprint performance, and agility in the experimental group compared with the control group.
Udiyapuram et al. (2025) [16]	Yoga Asana Group vs. Control (12 weeks)	Cardiovascular Endurance (VO2 max); Muscular Strength (Push-ups); Muscular Endurance (Sit-ups); Flexibility	Yoga: CVE 28.96 ± 2.03; MS 19.04 ± 1.39; ME 22.61 ± 2.17; FLE 22.33 ± 1.52; Control: similar baseline values	Yoga: CVE 31.28 ± 1.26; MS 20.66 ± 1.65; ME 25.19 ± 1.12; FLE 24.52 ± 1.03; Control: minimal changes	Yoga: CVE +2.12; MS +1.62; ME +2.58; FLE +2.19; Control: negligible changes	Significant improvements in yoga group (*p* < 0.001); no significant changes in control (*p* > 0.05)	CVE d = 1.18; MS d = 0.92; ME d = 1.26; FLE d = 2.51 (large effects)	↑ Endurance, strength, flexibility (yoga group superior)

Note: The symbols (+) and (−) indicate the direction of change from pre- to post-intervention, representing increases and decreases, respectively. Arrows (↑/↓) are used to denote improvement depending on the nature of the variable, where an increase (↑) reflects improvement in performance-related outcomes (e.g., strength, flexibility), and a decrease (↓) reflects improvement in reduction-based outcomes (e.g., stress, BMI, time).

## Data Availability

No new datasets were generated or analyzed in this study. All relevant information and original contributions are included within the manuscript; therefore, data sharing is not applicable.

## Supplementary Materials

The following supporting information can be downloaded at: https://www.mdpi.com/article/10.3390/healthcare14131924/s1, Table S1. Categories of Full-Text Articles Excluded During Eligibility Assessment and Reasons for Exclusion. Table S2. Narrative Certainty of Evidence Assessment Based on GRADE Principles.
