# Supplementary material for: Multidimensional Effects of Suryanamaskar on Physical, Physiological, and Psychological Outcomes: A Systematic Review
Source: Healthcare (Basel). 2026 Jul 1;14(13):1924. doi: 10.3390/healthcare14131924 (PMC13362503; doi:10.3390/healthcare14131924)
Supplement: Supplementary file 1 [file healthcare-14-01924-s001.zip › healthcare-4336769-supplementary.pdf]

Supplementary Table S1. Categories of Full-Text Articles Excluded During Eligibility Assessment and Reasons for Exclusion

Study/Study Description	Primary Focus	Reason for Exclusion
Slow versus Fast Suryanamaskar studies	Comparison of physiological responses to different speeds of Suryanamaskar practice	Acute intervention design and intervention duration did not satisfy the predefined eligibility criteria for structured intervention studies.
Acute cardiovascular and metabolic response studies	Immediate cardiovascular and metabolic responses following a single session of Suryanamaskar	Acute laboratory studies outside the scope of sustained intervention effects examined in the present review.
Acute cardiorespiratory and cognitive response studies	Immediate cardiorespiratory and cognitive responses following a single bout of Suryanamaskar	Acute experimental studies not designed to evaluate chronic intervention-induced adaptations.
Psychological outcome studies in adolescents not meeting PICOS criteria	Psychological responses associated with yoga or Suryanamaskar-related practice in adolescents	Outcome and/or study-design characteristics did not satisfy the predefined PICOS eligibility criteria.
Additional full-text records identified during eligibility assessment	Various Suryanamaskar-related topics	Excluded because of one or more predefined criteria, including ineligible intervention duration, inappropriate study design, absence of an eligible Suryanamaskar-based intervention, non-quantitative outcomes, or insufficient data for extraction.

Note: PICOS = Population, Intervention, Comparator, Outcomes, and Study Design. Full-text articles were excluded only after detailed eligibility assessment according to the predefined PICOS criteria and review protocol. Studies investigating acute responses to a single session of Suryanamaskar were excluded because the present review focused on intervention studies examining multidimensional adaptations following structured Suryanamaskar-based programmes.

Supplementary Table S2. Narrative Certainty of Evidence Assessment Based on GRADE Principles

Outcome Domain	Number of Studies	Primary Reasons for Downgrading	Overall Certainty of Evidence
Physical Fitness	9	Some concerns or serious risk of bias in several studies; substantial heterogeneity in intervention protocols, participant characteristics, and outcome measures; relatively small sample sizes	Low
Physiological Parameters	5	Methodological limitations, heterogeneity in physiological outcomes and intervention	Low

		characteristics, and imprecision due to small sample sizes	
Psychological Outcomes	6	Serious risk of bias in several studies, heterogeneous outcome measures (e.g., stress, depression, resilience, emotional intelligence), indirectness, and imprecision	Very Low
Body Composition	4	Limited number of studies, serious risk of bias, substantial heterogeneity in interventions and outcome assessment methods, and small sample sizes	Very Low

Note: Certainty ratings were determined narratively according to GRADE principles by considering risk of bias, inconsistency, indirectness, imprecision, and potential publication bias. Evidence was downgraded primarily because of methodological limitations of the included studies, substantial heterogeneity in intervention protocols and outcome assessments, relatively small sample sizes, and the inclusion of several non-randomized and self-controlled study designs. Formal evidence profiles and pooled effect estimates were not generated because considerable methodological and clinical heterogeneity precluded quantitative synthesis.
